# The Impact of Leader Humor on Employee Creativity during the COVID-19 Period: The Roles of Perceived Workload and Occupational Coping Self-Efficacy

**DOI:** 10.3390/bs13040303

**Published:** 2023-04-03

**Authors:** Lili Hu, Long Ye, Ming Guo, Yunshuo Liu

**Affiliations:** School of Economics and Management, Beijing Jiaotong University, Beijing 100044, China

**Keywords:** leader humor, employee creativity, perceived workload, occupational coping self-efficacy, similarity perception, Chinese employee

## Abstract

Based on the relief theory and similarity attraction theory, this study investigates the influence of leader humor on employee creativity through the mediate impact of employees’ perceived workload, occupational coping self-efficacy, and employee similarity perception with a leader as a potential moderator. The data were collected through an online survey that included matched questionnaire data from 351 employees and their direct leaders in China. This study used SPSS 26 software and Mplus 7.0 software to analyze the data and found that (1) leader humor has a significant positive impact on employees’ creativity; (2) employees’ perceived workload and occupational coping self-efficacy mediated the positive relationship between leader humor and employee creativity; (3) similarity perception negatively moderated the influence of leader humor on perceived workload, and it also positively moderated the influence of leader humor on occupational coping self-efficacy. In addition to corroborating and expanding on previous findings regarding the relationship between leader humor and employee creativity during the COVID-19 period, the aforementioned conclusions also derive management implications for fostering employee creativity and reducing employee workload from the perspective of leader humor.

## 1. Introduction

The COVID-19 pandemic has created global havoc, affecting almost all countries that touched the aspect of our lives, including family, education, health, work, and the relationship between leaders and followers in our society [1,2]. Amid the COVID-19 pandemic, and employees’ perceived workload has increased due to layoffs, downsizings, and job and financial insecurity [3]. Leadership and creativity are key components of the company’s response to the economic challenges of the COVID-19 pandemic. In addition, the pandemic has led to long-term emotional trauma and disrupted the psychological state of employees, which could have a negative impact on the sustainability of the company [4]. In this situation, it is more necessary to consider organization leaders’ relief role in coping stress and enhancing employees’ confidence in work, since leaders adapt to work on new realities [5].

According to scholars and practitioners, humor is a valuable coping technique for managers [6], which can help employees cope such problems positively by lessening their perceived workload, increasing a sense of control over any stress [7]. Several political and business leaders have undergone humor training to improve their leadership effectiveness [8]. Organizational researchers have specifically examined the impact of leader humor on both employees’ affective states and work outcomes [9,10,11]. Previous studies were mainly rooted in the daily stable work situation, and there is still a lack of detailed answers about the motivating factors of employees’ creativity under public health emergencies (such as the COVID-19 pandemic), more investigation and verification are required. Leader humor can significantly smooth the negative impact of COVID-19 [12]. Furthermore, prior studies have indicated that encouraging creativity and inventive employee behavior is an effective method for businesses to obtain and sustain competitive advantages [13]. Research focus on the influence factors of employees’ creativity includes employees’ behaviors, motivation, personality [14,15] and leadership styles [16]. Meanwhile, it is difficult to eliminate all the pressures in the working environment and, researchers have asserted that positive cognition is significant related to individual creativity [17]. Humor is a valuable workplace behavior and a helpful element for managers [18], which may help employees feel relieved and contribute to their confidence [19], experience positive emotions [20]. The relief theory proposed that humor can get people in a more relaxed mood which causes them more receptive to changes [21]. Interestingly, although relief theory was the “oldest” framework, there was much less research applying the relief theory of humor in explaining the effects of leader humor [22]. Considering humor as effective coping strategy for dealing with stress [23], this study attempts to explore the mechanism for leader humor to promote employee innovative behavior during the COVID-19 pandemic.

Relief theory holds that leader humor can affect individual behaviors through creating emotional relief and making employees feel safe with the situations [21]. Specifically, leader humor can be used to create a link with employees. Previous scholars have claimed that when employees perceive humor from their leaders, they would be change their view of the stressors such that they see the pressure as benign and recover a sense of control over any stress [7], and making employees more confident in creative problem-solving [24,25]. As important parts of individual self-concept, occupational coping self-efficacy is individual’s perception of ability to cope with job demands [26], which is essential for employee creativity and innovative behavior. Besides, Benhamou and Piedra [27] proposed that employees may suffer from more work load when they working for essential works during the COVID-19. This critical problem has becoming increasingly salient and frequent [24] under the impact of COVID-19, while the negative influence of stress on the employees’ innovative behavior in the workplace have been observed by the scholars [7,28]. Meanwhile, it is difficult to eliminate all the stressors in essential industry during the COVID-19 and, researchers have asserted that employees might respond differently when faced with the same stressful environment depending on their characteristics and organization context [29]. They might deal with stress positively by utilizing occupational self-efficacy and leader’ support. Similarly, this study also attempts to introduce perceived workload and occupation coping self-efficacy to explore the internal mechanism for leader humor to promote employee creativity.

Academics call for organizational management research to be carried out in a specific context [25]. One of the most important implicit hypotheses about the leader humor effectiveness is that divergent expectations and value sets during leader–employee interactions, employees can understand the intention and information of leaders using humor in the mature phase rather than early phase [30]. Similarity attraction theory holds that individuals more easily interact and socially connect if they share common personal characteristics [31]. According to the similarity attraction theory, similarity in characteristics such as personality, attitudes, and values influences attraction, leaders and followers who were similar on the predictor variables had higher quality relationships [32], leader-member exchange quality [33], workplace energy, and improve the team’s performance [34]. A meta-analysis of over 300 similarity studies also observed that similarity produces a positive, moderately sized effect on attraction [35]. The use of humor is increasingly encouraged by both practitioners and scholars [36], it is critical to examine whether similarity in leader-employees relationships is better than dissimilarity. Hence, this study attempts to explore employees similarity perception with leaders as a moderator in the relationships between leader humor and employee perceived workload and occupation self-efficacy.

**Figure 1:**
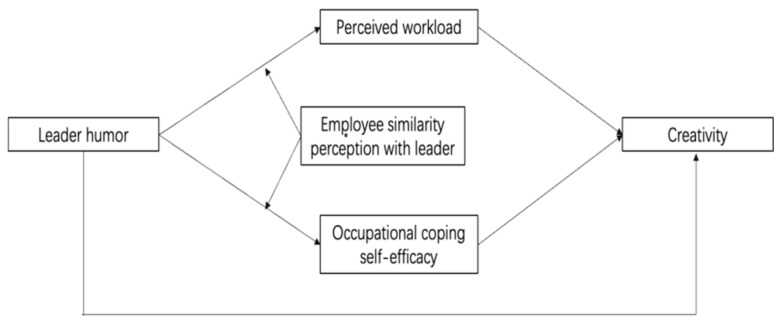
Hypothetical Model.

This study makes several contributions to understanding the importance of leader humor during the COVID-19. First, considering the global impact of COVID-19 in workplace, knowing how to properly use leadership to communicate with employees to promote creativity is paramount. Second, uncertainties and risks related to work status and health can inevitably cause emotional distress [37]. Employees must be creative under varying stress levels during a crisis like COVID-19. Still, organizational research has neglected the essential role of workload relief [11]. Therefore, this study provides a unique perspective on how leader’s humor use weaken employees’ workload perception and enhance occupational coping self-efficacy, and elucidates humor influence mechanisms and expounds on the stress relief functions of humor [11]. It also examines the effects of multiple mediations in leadership studies [38]. Third, the relationship between leader humor and employee outcomes may exhibit opposite consequences in different contexts. The similarity between leaders and employees should be considered to understand the effects of leader humor on employee creativity. The effects of leader humor on employee creativity may vary depending on the level of similarity perception between them. Figure 1 shows the study proposed model.

## 2. Theoretical Background

### 2.1. The Direct Effect of Leader Humor on Employees Creativity

Leader humor is a critical interpersonal resource that leaders can use to motivate subordinates to voluntarily engage in behaviors that directly or indirectly benefit them [11]. Employee creativity is defined as any idea and act that extends beyond the existing work standards or procedures in order to provide better service production or delivery [14]. As a powerful form of coping technique, humor is intended to be amusing in social communication [39], enhancing workplace outcomes, such as subordinate organization citizenship behaviors and leader–member exchange [11], performance, and creativity [40], and relieving potential work stress [11]. This multisource research has examined the importance of humor to employees, leaders, and organizations. Following earlier research, this study concludes that leader humor may successfully inspire employee creative behavior for the two reasons listed below:

First, the relief theory of humor implies the function of the stress relief mechanism, and leader humor performs as an interpersonal resource for stress relie [11]. The relief theory focuses on the physiological release of tension by laughing and provides a tool to overcome restrained emotions [23]. Leader humor, as interpersonal behavior, is essential for employee creativity [41]. Leaders interact with subordinates using humor, conveying support and friendship [11], and encourage non-conventional approaches to routine matters through its playful orientation, thus opening new insights for exploration and development [42]. In a relaxed atmosphere, employees are more willing to exchange new ideas and try new strategies. Leaders who are good at using humorous expressions are more willing to break the rules and accept employees’ behaviors outside the rules [43], thereby supporting employees’ innovative behaviors.

Second, during the COVID-19 pandemic, teleworking may lead to social or professional isolation [44], as well as social loneliness [45,46]. This feeling adversely affects job stress, and satisfaction [45], as well as burnout [46]. Research found that affiliative humor strengthened the negative effect of leader sense of humor on the workplace loneliness climate and resulted in better team performance [47]. Meanwhile, COVID-19 influences employee job insecurity, which, in turn, affects employee work and non-work outcomes (emotional exhaustion, organizational deviance, and saving behavior) [48], while affiliative humor buffers the relationships of both quantitative and qualitative job insecurity with burnout [49]. Only a few studies have addressed the role of humor during the COVID-19 crisis. For instance, the use of humor was found to be associated with psychological well-being during COVID 19 among individuals with a chronic illness and disability [50]. Leader humor can significantly smooth the negative impact of COVID-19, which can help improve employees’ positive psychological states and improve their initiative deviant innovation behavior [12]. Besides, leader humor can facilitate a high-quality relationship between supervisors and subordinates [11,51]. In order to retain this cordial connection with their superiors, employees will strive harder to solve work-related difficulties, actively generate new ideas, and seek inventive methods to improve their work processes. We suppose that leader humor expression facilitates employees to feel the trust and support from leaders, which will reduce the sense of insecurity caused by the COVID-19, and optimistic employees will continue to work hard under uncertain employment situations [52]. Based on theoretical arguments and previous empirical findings, we proposed that

**Hypothesis** **1.** *Leader humor is positively correlated with employee creativity*.

### 2.2. Perceived Workload as a Mediator between Leader Humor and Employee Creativity

Perceived workload(referred to as workload) is the perception that one has too much work to do [53], which negatively related to several job performance dimensions [54], and positively related to nursers intention to leave [55], physician burnout [56], stress and burnout [24], physical and psychological stress [57]. Therefore, we regard it as representing a threat to one’s resources. Besides, studies shown that humor helps people deal with job stress, such as subordinate stress experiences [58], subordinate burnout [59] and stress- related outcomes associated with coping [18,24]. Furthermore, given that work involves stress, humor can help individuals re-appraise or directly alleviate work stress [11], create a relaxed and pleasant organizational atmosphere [42]. Humor used in the workplace also helps employees with social support to relieve stressful and depressed situations [60]. When leaders leverage humor to share interesting things with their employees, the letter can personally feel relax and support from their leaders [61], this helps them relieve their perceived workload.

As a dynamic and intricate process, innovation requires a variety of tries and mistakes, as well as ongoing improvements based on knowledge, skill, and drive [62]. Therefore, it requires individuals to commit enough resources [63]. The perceived workload is the pressure associated with job demands [54]. It negatively correlates with employee emotional exhaustion and service performance [64]. Based on relief theory, when employees perceive their workload as unmanageable, they may interpret it as negative information that triggers adverse emotions such as anger, fear, and frustration [65]. It may hinder employee growth opportunities [66] and withdrawal from the ongoing situation. Stressful challenges that vary across periods negatively impact employee performance and well-being [67]. In a word, if employees don’t have enough resource to face various problems at work, their creativity will be constrained. Perceived workload consumes or occupies cognitive resources, this should undermine creativity [68]. Employees with low perceived workload can fully mobilize the resources around them to meet the challenge encountered in their jobs. As a result, they are more likely to produce creative ideas at work and subsequently seek methods to implement them, so fostering innovation.

According to the relief theory, the relaxed and humorous information reassures employees in times of heavy workload, and there may even be some emotional contagion effects [69]. Consequently, according to Cooper, Kong [11], we expect leader humor, associated with a relaxed, humorous expression, to help employees alleviate the perceived workload, reassure subordinates and allow them to deploy their resources effectively because employees’ resources outweigh work demands and are less likely to consider workload threatening. When employees’ perceived workload is relieved, they will become more tolerant, patient, active, and innovative to solve customers’ problems. Therefore, this study proposes that perceived workload would be a mediator in the relationship between leader humor and employee creativity, as follows:

**Hypothesis** **2.** *Perceived workload plays a mediating role between leader humor and employee creativity*.

### 2.3. Occupational Coping Self-Efficacy as a Mediator between Leader Humor and Employee Creativity

Self-efficacy is an individual’s confidence [70] or cognition of ability [71] in completing his work. Given that self-efficacy reflects a person’s perception of particular behaviors, the concept is situation-specific [72] and provides greater explanatory power, such as employee coping self-efficacy in the workplace, firefighters coping self-efficacy, and nurses coping self-efficacy in facing stressful and traumatic experiences encountered [73]. The individual may show high self-efficacy in one situation but low self- efficacy in another [74]. Hence, assessing context-specific rather than general self-efficacy perceptions is critical. Occupational coping self-efficacy is a specific type of self-efficacy separated from job-related self-efficacy [75], which focuses on an individual’s perception of ability to cope with job demands [26] and certain work stressor. Specifically, the idea of occupational coping self-efficacy for employees include a self-efficacy feature pertaining to their conviction in their abilities to manage interpersonal relationships and workplace problems [76]. Employees with high levels of coping self-efficacy are more likely to regard job demands as pleasant and challenging experiences, which impacts their motivation to persevere and invest effort in overcoming these challenges [75]. As a work-related intrapersonal resource [26], employees with higher levels of occupational coping self- efficacy were associated with lower levels of distress [77], which is critical for employees during the COVID-19 period.

Prior research has revealed that leaders can make a difference in facilitating employees’ coping with stress by creating environments that promote self-confidence, reducing stress and potential burnout [26]. Besides, leaders’ humorous expression sends recognition and support signals to their subordinates [78]. It can also stimulate employees’ positive emotions and make them stable and happy, significantly improving their self- efficacy [41]. Scholars believe that occupational coping self-efficacy is a significant predictor of employees’ cognition [79] in uncertain or stressful situations. Employees with high self-efficacy believe that they can effectively complete tasks and objectives and generate more innovative behaviors [80]. Improvement in employees’ self-efficacy enhances employee creative performance over time [81]. Individuals with high self- efficacy can actively and continuously perform innovative activities.

Besides, several studies reported that employees’ self-efficacy plays a mediating role between leadership and employee creativity, such as supervisor expectation and behaviors [82], creativity role identity [83], positive leaders’ implicit followership theory [84]. Leaders convey a relaxed, harmonious climate in the workplace through humorous expressions, stimulating employees’ positive emotions to make them stable and happy [41], increasing their confidence in work. Employees’ self-efficacy enhances the generation and acceptance of new ideas and behaviors [85]. Therefore, this study proposes that occupational coping self-efficacy would be a mediator in the relationship between leader humor and employee creativity, as follows:

**Hypothesis** **3.** *Occupational coping self-efficacy plays a mediating role between leader humor and employee creativity*.

### 2.4. Moderating Effect of Similarity Perception

The similarity attraction theory holds that individuals more easily interact and socially connect if they share common personal characteristics [31]. Similarity perception can enhances or weakens the influence of leaders on their employees’ growth-need strength [86] and job performance [87], whether in the early or mature stage during leader– member interactions. The similarity between leaders and employees helps ease interpersonal communication [88]. When a leader and a follower share similarities, their relationship is more positive [89] and more likely had higher quality relationships [32], leader-memberexchange quality [33], workplace energy, and improve the team’s performance [34]. As the research work go deep, some scholars begin to pay attention to the moderating role of similarity perception between leader and employees amplifies the impact of leaders on employees. For example, Tan, Wang [87] indicated that the genders similarity of the leader and subordinates could moderate the relationship between leader humor behavior and employee job performance. Perceived mentor and newcomer deep similarity can moderate the relationship between newcomers’ relationship-building behavior and mentor information sharing, person-supervisor deep-level similarity significantly interact with job insecurity to predict job satisfaction [90]. A meta-analysis of over 300 similarity studies also observed that similarity produces a positive, moderately sized effect on attraction [35].

According to the similarity attraction theory, employees with a high similarity perception with leaders show high humor appreciation, which can help them obtain positive emotional information from leaders’ humor, reducing their workload and increasing confidence in creative work. Humor appreciation relies on the ability of employees to adopt new perspectives quickly [91]. On the contrary, when employees have low similarity with their leader, more tension may be experienced due to uncertainty about the leader’s attitude in a virtual environment. To be specific, for employees with high similarity perception with leaders, leader humor would have greater impact on their occupation self—efficacy and alleviate employees’ degree of perceived workload. In contrast, employees with low similarity perception with leaders are less likely to be affected by the humor of their leaders and less likely to actively improve their occupation self-efficacy and reduce perceived workload. Hence, we propose that:

**Hypothesis** **4a.** *Similarity perception moderates the negative relationship between leader humor and workload such that the relationship is higher when similarity perception is lower*.

**Hypothesis** **4b.** *Similarity perception moderates the positive relationship between leader humor and occupational coping self-efficacy such that the relationship is stronger when similarity perception is higher*.

## 3. Materials and Methods

### 3.1. Sample and Data Collection

A questionnaire survey was conducted to test our hypotheses. We contacted 10 hotels that were performing the task of hotel isolation at that time, and 5 hotels responded and expressed their willingness to cooperate with this survey. In sample selection, we selected front-line employees and managers who were working in the hotels at that time, which were located in Beijing, Shanghai, Wuhan, and other eastern China cities. These employees were directly exposed to the working environment with the possibility of COVID-19. We contacted the departmental managers of these hotels prior to collecting the questionnaire data and explained that this survey was for academic purposes. It did not cause any adverse effects on employees or their companies. Any questionnaire information will not be disclosed to other participants.

Data collection was accomplished through a web-based and paper questionnaire survey. The survey was divided into two stages. At time 1, the employees were required to fill in the questionnaire via email to measure their perception of leader humor and similarity perception, as well as their demographic information (e.g., gender, age, work tenure with their direct leader, education). We obtained 400 valid responses. At time 2, the second survey was conducted 1 month later. Employees completed the workload perception and occupational coping self-efficacy questionnaire, and their direct leaders evaluated employees’ creativity. In the second round, 351 employee–leader dyads’ valid responses were obtained, with an effective recovery rate of 87.75%. The final employee sample consisted of 240 female (68.4%) and 111 male (31.6%) staff. Of course, they were mainly aged between 25 and 40 years of age (95.12% of the total study participants). Most respondents were under 5 years of tenure (49.6%), and 41.6% had a college degree. For leaders, the final leader sample consisted of 24 female (32.9%) and 49 male (67.1%), and they were mainly aged 35–45 years of age (80.8%), with most of them having a university degree (81.6%).

### 3.2. Measures

In academic research, the scales used are widely used authoritative representative scales. Since this study was performed in China, the original English version questionnaire was translated into Chinese following the translation committee approach [92] to ensure that the scale is as close to the original scale as possible. Three researchers from human resources and English majors were invited to do the revisions. The revisions included clarifying certain words of several items, and changing the questionnaire format, thereby ensuring the equivalence of meaning between the English and Chinese versions. Likert 7 points were used to score the scales. From “1” to “7”, the degree of conformity to the item’s description was from low to high.

Leader humor: The three-item leader humor measure of Cooper, Kong [11] was used to measure employees’ perception of humor from their direct leader, items such as “I think a leader is a witty person.” The Cronbach’s 𝛼 was 0.864.

Occupational coping self-efficacy: this measure adapts the 9-item Occupational Coping Self-Efficacy Questionnaire [76], which assesses an individual’s confidence in his/her ability to cope effectively with COVID-19 quarantine service work in the hotel. The word ‘patients’ at each item of the original measure was changed to ‘hotel customers.’ Examples of scale items were as follows: ‘difficulties with customers’ ‘difficulties in deciding how to do the work.’ The Cronbach’s 𝛼 was 0.883.

Perceived workload: The 5-item scale measured the employees’ perceived workload [93]. Items such as “My work requires quick completion” were measured. The Cronbach’s 𝛼 was 0.636.

Creativity: A 4-item scale specially developed for Chinese creativity research is widely adopted in many researches [94], with sample questions such as “He will try some new ideas or methods at work”. In this paper, leaders evaluate the creativity of employees, and the Cronbach ‘S value was 0.917.

Similarity perception: A 6-item scale developed by Lankau was used to measure similarity perception [95]. Items such as “I am similar to leaders in problem-solving methods” were measured. The Cronbach’s 𝛼 was 0.910.

Control variables: Considering this research explores the mechanism of leader humor and employee creativity, and age, gender, and education have been shown to be associated with creativity [96], this study consistent with previous studies [7,11,25], selected gender, age, tenure, and education level as the main control variables at Time 1.

## 4. Results

### 4.1. Reliability and Validity Tests

#### 4.1.1. Reliability Test

The reliability of leader humor, workload, occupational coping self-efficacy, creativity, and similarity perception was determined using SPSS 26. Cronbach’s 𝛼 for workload was lower than 0.7, while for the other four variables, it was higher than 0.7. We tested the aggregation and discrimination validity to ensure the rationality and reliability of the measurement scale selected by the sample [97].

#### 4.1.2. Aggregation Validity Test

This study calculated the average variance extracted (AVE) for each variable using each item’s load factor coefficient to measure its aggregation validity (Table 1). Each variable AVE is more significant than 0.5, indicating a good aggregation validity [98].

#### 4.1.3. Distinguishing Validity Test

Employees evaluated leader humor, workload, occupational coping self-efficacy, and creativity. We used a five-step procedure to assess data structure [99]. First, Mplus7.0 was used to perform a series of confirmatory factor analyses (CFA) on leader humor, similarity perception, workload, occupational coping self-efficacy, and creativity constructs, which tested whether the measurement model in this study had a better fitting degree (Table 1). We constructed a model with these five factors, and the fit indices were acceptable (λ2/Df (282) = 1.652, TLI = 0.946, CFI = 0.956, SRMR = 0.051, RMSEA = 0.048), which implied that the five-factor model provided a good fit that was better than those of the other models. The CFI is slightly higher than 0.90, which is the value typically considered as evidence of good fit [100]. Similarly, RMSEA was 0.048, which falls within the cut-off points, indicating acceptable model fit. It shows that the five factors have good discriminant validity and are different constructs.

### 4.2. Descriptive Results

The mean values, standard deviations, and correlation among all variables are shown in Table 2. There was a significant positive correlation between leader humor and employee workload, as well as employee occupational coping self-efficacy and creativity. As expected, leader humor was positively correlated with employee creativity (γ = 0. 406, *p* < 0.01) and occupational coping self-efficacy (γ = 0.259, *p* < 0.01), negatively correlated with workload (γ = −0.207, *p* < 0.01), and occupational coping self-efficacy was positively correlated with employee creativity (γ = 0. 431, *p* < 0.01). In contrast, the workload was negatively correlated with employee creativity (γ = −0.577, *p* < 0.01). These correlations provide an intuitive impression for subsequent regression analysis and data analysis.

Furthermore, a multicollinearity test showed that the highest variance inflation factor (VIF) was 1.972, while the lowest tolerance value was 0.507. VIF values were less than 10, while tolerance values were higher than 0.10. Therefore, multicollinearity was not a significant issue in this study.

### 4.3. Hypothesis Tests

#### 4.3.1. Main Effect Analysis of Leader Humor on Employee Creativity

Hierarchical regression analysis was used to test the relationship between leader humor, perceived workload, occupational coping self-efficacy, similarity perception, and creativity. Table 3 shows that, after controlling for employees’ gender, age, tenure, and education level, model 5 indicates that leader humor is positively related to employee creativity (β = 0.285, *p* < 0.01), so Hypothesis 1 is supported.

#### 4.3.2. Mediate Effects Analysis of Leader Humor on Employee Creativity

This study included two mediators. For the mediating effect of perceived workload and occupational coping self-efficacy, this study followed the procedures proposed by Preacher and Hayes [101] to test the indirect influence of leader humor on employee creativity via perceived workload and occupational coping self-efficacy. As shown by model 5,6, leader humor was a significant direct predictor of employee creativity (Model 5: β = 0.285, *p* < 0.01). After adding perceived workload and occupational coping self- efficacy to the hierarchical regression analysis model, perceived workload (Model 6: β = −0.244, *p* < 0.01) and occupational coping self-efficacy (Model 6: β = 0.533, *p* < 0.01) could also significantly predict employee creativity; meanwhile, the influence of leader humor on employee creativity is still significant (Model 6: β = 0.090, *p* < 0.01), suggesting that perceived workload and occupational coping self-efficacy could partly mediate the influence of leader humor on employee creativity.

In order to analyze the indirect effect that leader humor has on employee creativity through perceived workload and occupational coping self-efficacy, this study used Bootstrap methods in virtue of PROCESS macros with Model 4. As shown in Table 4, the impact of leader humor on creativity is partially confirmed through two mediating variables. The total effect of leader humor on employee creativity is significant (β = 0.285, *p* < 0.01), the total indirect effect accounts for 68.42% of the total effect of leader humor on creativity, and the 95% confidence interval of bootstrap is (0.222, 0.349) excluding 0, which indicating that hypothesis 1 was supported again. Besides, the results showed that the indirect influence of leader humor on employee creativity through perceived workload is significant (indirect effect = 0.034, with a 95% CI of [0.016, 0.083]). Hypothesis 2 is, therefore, well supported. Finally, the indirect influence of leader humor on employee creativity through occupational coping self-efficacy is significant (indirect effect = 0.161, with a 95% CI of [0.106, 0.306]). Hypothesis 3 is, therefore, well supported. Due to the (CI), we can see that occupational coping self-efficacy plays a higher mediating effect than workload in the dual mediating path of leader humor impacting employee creativity (effect = −0.127, with a 95% CI of [−0.227, −0.079]).

#### 4.3.3. Moderation Mechanism Test

For the moderating effects of similarity perception in the relationship between leader humor, perceived workload, and occupational coping self-efficacy, this study adopted Hayes [102] procedures for testing a moderating effect. After control over employees’ gender, age, tenure, and education, as with Models 1, 2, 3, and 4, leader humor also becomes a significant predictor of employee perceived workload (Model 1: β = −0.141, *p* < 0.001) and occupational coping self-efficacy (Model 3: β = 0.196, *p* < 0.001). Meanwhile, the interaction term of leader humor and similarity perception is significant in predicting perceived workload (Model 2: β = −0.100, *p* < 0.005) and occupational coping self-efficacy (Model 4: β = 0.097, *p* < 0.005).

**Figure 2:**
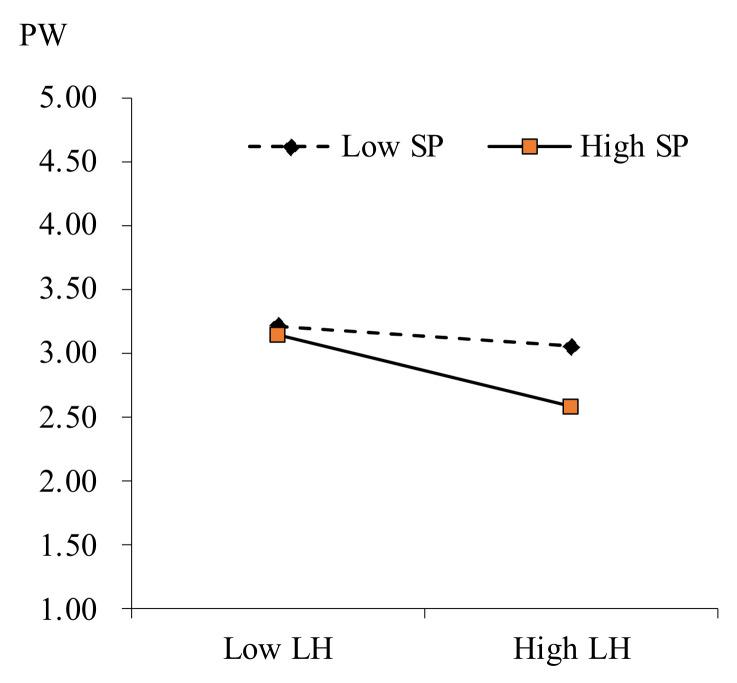
Moderated effect of similarity perception in the relationship between leader humor and perceived workload.

**Figure 3:**
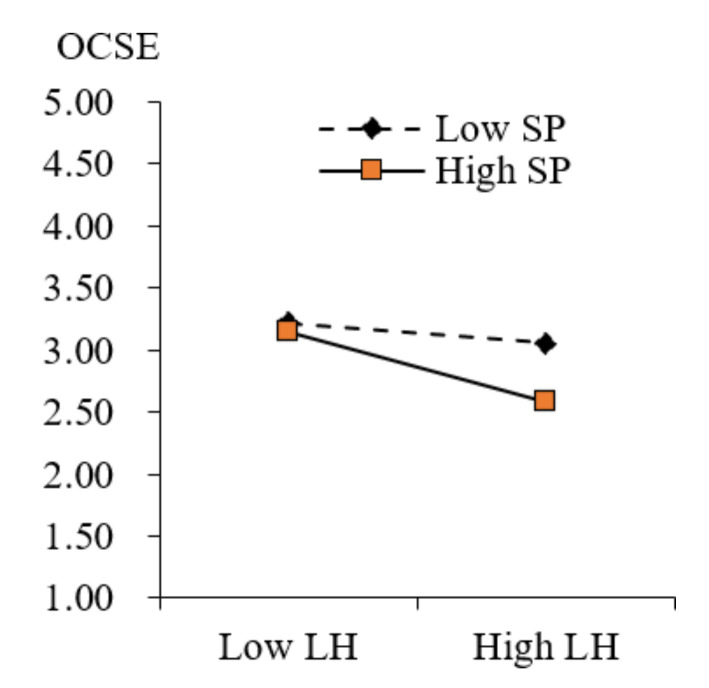
Moderated effect of similarity perception in the relationship between leader humor and occupational coping self-efficacy.

Further, this study plot this interaction as a conditional value of similarity perception (one standard deviation above and below the mean), as displayed in Figure 2 and Figure 3. From Table 5, the results confirm that the direct influence of leader humor on perceived workload is significant for employees with high similarity perception (b = 0.271, 95% CI = [0.051, 0.490]; +1 SD) but not for employees with low similarity perception (b = −0.008, 95% CI = [−0.195, 0.210]; −1 SD). Hence, Hypothesis 4a is well supported.

Finally, as for the interaction of leader humor and similarity perception on occupational coping self-efficacy, the results confirm that the direct influence of leader humor on occupational coping self-efficacy is significant for employees with high similarity perception (b = 0.530, 95% CI = [0.350, 0.710]; +1 SD) and low similarity perception (b = 0.283, 95% CI = [0.118, 0.449]; −1 SD). Hence, Hypothesis 4a is well supported.

## 5. Discussion

Leader humor is a useful coping strategy in managing stress and interpersonal communication. Previous studies have addressed the relationship between leader humor and creativity or innovation of employees [25]. Using matched questionnaire data from 351 employees and their direct leaders in China, this study found that leader humor has a significant positive impact on employees’ creativity, and employees’ perceived workload and occupational coping self-efficacy partly mediated this relationship. In addition, similarity perception negatively moderated the influence of leader humor on perceived workload and positively moderated the influence of leader humor on occupational coping self-efficacy.

### 5.1. Theoretical Implications

The findings of this study contribute to the literature on humor and creativity in several ways. First, this study suggests that leaders have a positive effect on employee creativity. Although some prior studies have attempted to explore the relationship between leader humor and individual outcome variables, the understanding of leader humor as a relief mechanism in academic circles is still very limited. Based on the samples of Chinese employees and their direct supervisors, this study reveals that leader humor can promote employee creativity. It would enrich the existing literature on leadership and innovation.

Second, we developed a conceptual framework to explain the humor–creativity relationship using relief and similarity attraction theories. Contrary to Cooper’s expectation, the stress relief explanation of LH was not supported [11]. Considering this gap and the culture differences between western culture and Chinese context, it is very important to explore the influencing mechanism of leader humor from different theoretical perspectives. Inspired by this idea, based on the humor relief theory, we take job characteristics (perceived workload and occupational coping self-efficacy) into a new mediating mechanism that enhances employee creativity through leader humor. Consistent with the humor relief theory, our findings show that perceived workload and occupational coping self-efficacy are central components linking leader humor to creativity. Leader humor is an efficient tool to alleviate employees’ perceived workload, which amuses employees in relaxed communication and gives them more support emotionally [43], reducing employees’ perceived workload and worries about tasks and giving more confidence to the employees to try new ideas and complete work. These effects enhance employees’ creativity, which is a win-win situation for both individuals and organizations.

More importantly, occupational coping self-efficacy is mainly used in hospitals for nurses’ burnout and mental health [43], as well as job turnover intentions [79]. Similar to nurses, employees face stressors due to being exposed to the crisis during COVID-19. However, there are limited studies explore employees occupational coping self-efficacy in the general workplace rather than hospital. Hence, this study mid this gap to testify the mediate role of employees occupational coping self-efficacy between leadership and employee creativity. Just as the study demonstrated the ability of task-coping in reducing work withdrawal behavior for hospitality employees [18], occupational coping self- efficacy is an important capacity to cope with stressors and stress for employees exposed to the COVID-19 workplace, not only for nurses but for all essential employees working in other industries [27]. In addition, our findings enrich the humor relief theory literature in a Chinese context, and they also respond to the call of Mao, Chiang [103] and open the “black box” in the process of leader humor motivating employee creativity in different perspectives.

Finally, this study explores an important boundary condition for the relationship between leader humor, employees’ perceived workload, and occupational coping self- efficacy. Our findings showed that leader humor is more effective for high similarity perception employees. If employees perceive high similarity with the leader, they will fully receive leader humor intention and enhance their creativity by strengthening leader humor’s influence on workload and occupational coping self-efficacy. The findings of this study suggest that companies may benefit from taking similarity into account when attempting to build effective leader–follower relationships and teams. Similarity perception plays an essential moderating role in buffering the negative impact of leader humor on job demands and promoting job resources’ positive impact. This finding enriched the leader humor research by testifying on a necessary context condition and instructing leaders to express humor to employees.

### 5.2. Practical Implications

This study has some reference values for management practice. Firstly, by examining the effect of leader humor on employee creativity, this research offers companies and organizations a practical reference for better leveraging leader humor. Increasing spiritual demands, work disruption due to the COVID-19 pandemic mean that employees may suffer more general distress [104]. To some degree, leaders may develop an exciting and relax climate in their companies by using humor [11]. Therefore, managers should pay more attention to the humor effective in management practice and use humor as an effective cope technique. For instance, well-intentioned jokes might be utilized to motivate employee potential [13]. In addition, humor training for leaders is a worth human resource investment, which can adjust leadership styles and help them use more suitable humor expressions in daily work.

Second, the finding that perceived workload and occupational coping self-efficacy serve as mediators suggests that focusing on employees’ stress coping can elicit high- quality creativity in organizations. Perceived workload is negative related to behavioral stress during COVID-19 emergency [105], this study take advantage of humorous features to improve their coping with stressful situations. By reducing employees’ workload and cultivating occupational coping self-efficacy, organizations can offer frontline employees more supportive conditions to increase their job confidence. Organizations, for example, can create a relaxing interpersonal environment in which employees can unwind and have more resources to serve customers. Organizations can also consider various ways to give employees job resources and reduce job demand, such as using service robots and digital technology to reduce face-to-face contact with isolated customers [106], thus improving their occupational coping self-efficacy and reducing workload.

Third, our findings showed that influences of leader humor are stronger when employees have a high level of similarity perception with their leaders. This indicates that leader humor effective would be better for those more familiar with leaders. Hence, as a humor “sender,” leaders should consider whether the humor “receiver” can understand their humor intentions or not [11] and use leader humor accordingly. When employees are a high similarity perception with the leader, leaders use more humor to help these employees regain sufficient positive effect [107].

### 5.3. Limitations and Future Research

Considering how COVID-19 has significantly impacted the normal activities of the organization and led to long-term emotional trauma and disrupted the psychological state of employees [4], this study explored whether leader humor affects employees’ creativity through occupational coping self-efficacy and perceived workload of employees, as well as how leader humor enhances employees’ occupational coping self-efficacy and reduces employees’ perceived workload by assuming similarity perception between leader and employees as a moderator. The limitations in this study are mainly reflected in the following aspects: In terms of research methods, this study obtained survey data from employee self-report scale reports and leader evaluations. Although the leader evaluated employee creativity, sufficient leader information was not collected, given the difficulty and quality of data recovery. The interference of leader demographic variables in the research model cannot be ruled out. There are some limitations. Future studies should aim to obtain information from leaders and employees on-the-spot to validate our findings. Secondly, although this study reveals the positive effect and moderate mechanism of leader humor, only the context factors of employees’ cognition are considered. Future studies should determine whether employee personality factors (such as proactive personality) and organizational situations (such as leader humor style) can regulate the above-mentioned mediatory path. Finally, this study focused on the influence of leader humor on employees, ignoring its influence on the leaders themselves. When employees respond to leader humor, do leaders experience more positive emotions and use humor more frequently, or is it that, when leader humor is not understood and accepted by employees, leaders reduce humor use and even question their management methods? In order to answer the above questions, future research can explore the mechanisms of leader humor in leadership.

## 6. Conclusions

This paper responds to Israeli, Mohsin [108] suggestion that studies should investigate crisis management in diverse locations and contexts. The primary goal of our research was to apply stress relief and similarity attraction theories to the relationship between leader humor and creativity, as well as investigate the role of perceived workload and occupational coping self-efficacy as potential mediators. We especially explored the leader and employee similarity as a potential moderator. Using multi-time data from pairing samples of 351 Chinese employees and their immediate supervisors, this study reveals that leader humor is positively associated with employee creativity, and this relationship is partly mediated by perceived workload and occupational coping self-efficacy. In addition, as expected, similarity perception acts as a moderator in the relationship between leader humor and perceived workload (occupational coping self-efficacy). Interestingly, only in high similarity perception with leader and employees can leader humor weaken employees’ perception workload.

## Figures and Tables

**Table 1:** Confirmatory Factor Analysis Test Results of Variables (N = 351).

Model	λ2	Df	λ2/Df	TLI	CFI	SRMR	RMSEA
Five-factor model (LH, SP, PW, OCSE, CR)	259.335	157	1.652	0.946	0.956	0.051	0.048
Four-factor model 1(LH, SP, PW + OCSE, CR)	390.009	164	2.378	0.887	0.902	0.067	0.069
Four-factor model 2(LH, SP, PW, OCSE + CR)	453.307	164	2.764	0.855	0.875	0.070	0.078
Three-factor model (LH, SP + PW + OCSE, CR)	893.087	167	5.348	0.642	0.685	0.114	0.123
Two-factor model (LH, SP + PW + OCSE + CR)	952.288	169	5.635	0.618	0.661	0.117	0.127
Single factor model (LH + SP + PW + OCSE + CR)	1170.878	170	6.888	0.515	0.566	0.118	0.143

Note: LH = leader humor; PW = perceived workload; OCSE = occupational coping self-efficacy; CR = Creativity, SP = similarity perception.

**Table 2:** Descriptive statistical, correlation coefficient, and reliability coefficient among variances (N = 351).

Model	ME	SE	1	2	3	4	5	6	7	8	9
1. Gender	1.406	0.023	1								
2. Age	30.949	0.192	0.049	1							
3. Tenure	7.761	0.236	0.020	0.819 **	1						
4. Edu	3.002	0.028	−0.019	−0.320 **	−0.423 **	1					
5. LH	4.737	0.133	−0.098 *	−0.109 *	−0.127 **	0.104 *	0.832				
6. PW	2.915	0.055	−0. 008	0.110 *	0.155 **	−0.136 **	0-.207 **	0.537			
7. OCSE	4.727	0.714	−0.108 *	−0.100 *	−0.156 *	0.077	0.259 *	−0.196 *	0.858		
8. SP	4.436	0.036	−0.114 *	−0.102 *	−0.160 **	0.213 **	0.539 **	−0.313 *	0.311 **	0.790	
9. CR	5.557	0.057	−0.010	−0.169 **	−0.208 **	0.197 **	0.406 **	−0.577 **	0.431 **	0.567 **	0.728

Note: LH = leader humor; PW = perceived workload; OCSE = occupational coping self-efficacy; SP = similarity perception; CR = creativity. ** *p* < 0.01, * *p* < 0.05.

**Table 3:** Influence of leader humor on perceived workload, occupational coping self-efficacy, and creativity (N = 351).

Variable	PW		OCSE		CR	
	Model 1	Model 2	Model 3	Model 4	Model 5	Model 6
Control variable						
Gender	−0.022	−0.035	−141	−0.111	0.027	0.050
Age	−0.058	−0.025	0.161	0.138	−0.006	−0.107
Tenure	0.151	0.122	−0.218 *	−188	−115	0.069
Education	−0.078	−0.032	0.027	−0.004	0.115 *	0.037
Independent variable						
LH	−0.141 **	−0.181 **	0.196 **	0.038 *	0.285 **	0.090 **
Mediator Variable						
PW						−0.244 **
OCSE						0.533 **
Adjusting variable						
SP		−0.136 *		0.362 **		
Interactive Item						
LH * SP		−0.100 **		0.097 *		
R2	0.053	0.107	0.086	0.132	0.190	0.617
△R2	0.034	0.014	0.056	0.011	0.143	0.417
F	16.113 *	7.190 **	26.920 **	5.757 **	78.211 **	237.456 **

Note: LH = leader humor; PW = perceived workload; OCSE = occupational coping self-efficacy; SP = similarity perception; CR = creativity. N = 351, ** *p* < 0.01, * *p* < 0.05; The regression coefficient is non-standardized regression coefficient; F: Analysis of Variance.

**Table 4:** Total, direct, and indirect effects of leader humor on creativity.

Path	Effect Value	SE	95% Confidence Interval
The total effect of leader humor on creativity	0.285	0.032	[0.222, 0.349]
The direct effect of leader humor on creativity	0.090	0.024	[0.042, 0.138]
The indirect effect of leader humor on creativity			
Total indirect effect	0.195	0.065	[0.126, 0.383]
Perceived workload	0.034	0.018	[0.016, 0.083]
Occupational coping self-efficacy	0.161	0.051	[0.106, 0.306]
(C1)	−0.127	0.037	[−0.227, −0.079]

**Table 5:** Simple effect analysis of moderation effects.

Path	Perceived Workload			Occupational Coping Self-Efficacy		
	Effect Value	SE	95% CI	Effect Value	SE	95% CI
−1sd	0.008	0.103	[−0.195, 0.210]	0.283	0.084	[0.118, 0.449]
Mean	0.139	0.094	[−0.045, 0.324]	0.407	0.077	[0.256, 0.558]
+1sd	0.271	0.111	[0.051, 0.490]	0.530	0.091	[0.350, 0.710]

## Data Availability

Not applicable.
